# Remotely monitoring ecosystem respiration from various grasslands along a large-scale east–west transect across northern China

**DOI:** 10.1186/s13021-020-00141-8

**Published:** 2020-04-24

**Authors:** Xuguang Tang, Yanlian Zhou, Hengpeng Li, Li Yao, Zhi Ding, Mingguo Ma, Pujia Yu

**Affiliations:** 1 State Cultivation Base of Eco-agriculture for Southwest Mountainous Land, Southwest University, Chongqing, 400715 China; 2 Chongqing Jinfo Mountain Field Scientific Observation and Research Station for Karst Ecosystem (Southwest University), Ministry of Education, Chongqing, 400715 China; 3 International Institute for Earth System Science, Nanjing University, Nanjing, 210023 China; 4 Nanjing Institute of Geography and Limnology, Chinese Academy of Sciences, Nanjing, 210008 China

**Keywords:** Ecosystem respiration, Grassland, MODIS, Piecewise regression

## Abstract

**Background:**

Grassland ecosystems play an important role in the terrestrial carbon cycles through carbon emission by ecosystem respiration (*R*_*e*_) and carbon uptake by plant photosynthesis (GPP). Surprisingly, given *R*_*e*_ occupies a large component of annual carbon balance, rather less attention has been paid to developing the estimates of *R*_*e*_ compared to GPP.

**Results:**

Based on 11 flux sites over the diverse grassland ecosystems in northern China, this study examined the amounts of carbon released by *R*_*e*_ as well as the dominant environmental controls across temperate meadow steppe, typical steppe, desert steppe and alpine meadow, respectively. Multi-year mean *R*_*e*_ revealed relatively less CO_2_ emitted by the desert steppe in comparison with other grassland ecosystems. Meanwhile, C emissions of all grasslands were mainly controlled by the growing period. Correlation analysis revealed that apart from air and soil temperature, soil water content exerted a strong effect on the variability in *R*_*e*_, which implied the great potential to derive *R*_*e*_ using relevant remote sensing data. Then, these field-measured *R*_*e*_ data were up-scaled to large areas using time-series MODIS information and remote sensing-based piecewise regression models. These semi-empirical models appeared to work well with a small margin of error (*R*^*2*^ and RMSE ranged from 0.45 to 0.88 and from 0.21 to 0.69 g C m^−2^ d^−1^, respectively).

**Conclusions:**

Generally, the piecewise models from the growth period and dormant season performed better than model developed directly from the entire year. Moreover, the biases between annual mean *R*_*e*_ observations and the remotely-derived products were usually within 20%. Finally, the regional *R*_*e*_ emissions across northern China’s grasslands was approximately 100.66 Tg C in 2010, about 1/3 of carbon fixed from the MODIS GPP product. Specially, the desert steppe exhibited the highest ratio, followed by the temperate meadow steppe, typical steppe and alpine meadow. Therefore, this work provides a novel framework to accurately predict the spatio-temporal patterns of *R*_*e*_ over large areas, which can greatly reduce the uncertainties in global carbon estimates and climate projections.

## Background

Although the terrestrial biosphere absorbs nearly a quarter of anthropogenic CO_2_ emissions and plays a critical role in mitigating global climate warming, the land C sequestration potential of different ecosystems remains highly uncertain, leading to large uncertainties in future climate projections [1, 2]. During the past several decades, substantial advances have allowed for spatially continuous, long-term estimation of terrestrial gross primary productivity (GPP) based on the satellite remote sensing data, climate data and ecosystem models [3–6]. However, net ecosystem C budget depends upon the balance of C fixation through vegetation photosynthesis and C loss from ecosystem respiration (*R*_*e*_) [7, 8], which requires accurate estimates of not only GPP but also *R*_*e*_ across biomes.

Actually, terrestrial *R*_*e*_ from the biosphere to the atmosphere represents a large component of annual carbon budget, which even exceeds the amount of GPP [9, 10]. It has excited much interest in evaluating the balance between GPP and *R*_*e*_ on regional to global scales [11–13], because small fluctuations in either component caused by natural or human disturbances can ameliorate or exacerbate the buildup of CO_2_ in the atmosphere [14–16]. Particularly, *R*_*e*_ is sensitive to the environmental factors and is highly spatio-temporally heterogeneous across scales [17, 18], which made it far poorly understood owing to the complicated interactions among physical, chemical, and biological variables in the respiration processes, including autotrophic respiration (*R*_*a*_) from vegetation itself and heterotrophic respiration (*R*_*h*_) from diverse soil microbiota [19]. Therefore, accurate quantification of the C emissions through *R*_*e*_ is crucial to understand its effect on climate change and global carbon dynamics.

With the development of global FLUXNET community based the eddy covariance (EC) technique across terrestrial ecosystems, it has been possible to continuously monitor the seasonal and interannual variations of carbon fluxes between the biosphere and the atmosphere, which can be further divided into GPP and *R*_*e*_ according to the nighttime based [20] and daytime based flux-partitioning methods [21]. However, the in situ observations are generally implemented at field scale with low areal coverage (< 1 km^2^) and high cost of constructing and maintaining flux towers [22–24]. The availability of spatially continuous data of ecosystem properties and environmental variables important for *R*_*e*_ provides an alternative approach for the large-area estimates. Currently, how to upscale the field-measured data using remote sensing (RS) information is urgently needed for understanding regional and global patterns of ecosystem *R*_*e*_.

Several studies have recently been conducted to model the spatial distribution of soil respiration (*R*_*s*_) at alpine grasslands [25] and forests [26, 27] using the satellite-based products including land surface temperature (LST) and spectral vegetation index (NDVI or EVI) or leaf area index (LAI). By incorporating the terrain information, Berryman et al. [28] estimated *R*_*s*_ in a typical of the Southern Rocky Mountains with a coefficient of determination (*R*^*2*^) of 0.45. Jägermeyr et al. [29] firstly developed the models of global *R*_*e*_ according to forested and non-forested biomes. However, the classification system means that parameterization may not take into account the wide variety of ecosystems on the earth [31]. Ai et al. [17] also proposed an empirical yet physiologically based model with *R*^*2*^ and RMSE of 0.55 and 1.67 g C m^−2^ d^−1^ respectively, which is capable of retrieving the patterns in *R*_*e*_ at the global scale. But the model cannot be transferred well to specific ecosystems, and may particularly be inaccurate in hydrologically sensitive areas owing to lack of water index affecting *R*_*e*_. Nevertheless, previous studies have implied the significant correlations between grassland respiration and vegetation growth status [25], as well as the environmental variables such as LST [15, 17, 30] from the time-series satellite data.

Grasslands are the dominant landscape in China and account for 40% of the national land area. Geographically, approximately 78% of the grasslands in China exist in the northern temperate and alpine zones, constituting an integral part of the Eurasian grassland ecosystem [32, 33]. Due to their large carbon content, grasslands account for one-third of the global terrestrial carbon stock, second only to the forest ecosystem [34, 35], and play a key role in China’s terrestrial carbon cycle. Zhang et al. [5] found that temperate grasslands in northern China have the potential to sequester carbon, but the capacity of carbon sequestration relies on grassland types and local environmental conditions. Further analysis revealed that the water availability is the dominant environmental factor regulating the annual carbon budget [36]. Extreme climate events such as drought can significantly reduce the net carbon uptake of grasslands. Moreover, it is predicted that heat waves and droughts will become more frequent in the 21st century [37], which may lead to a general decrease in vegetation productivity in these grassland systems of northern China. Although several studies suggested that grasslands might be weak C sinks or near equilibrium [5, 38], as the main constraint on C budget, a deep understanding of *R*_*e*_ is helpful to project climate change-terrestrial C feedback over different grassland ecosystems. Specially, the goals of this study were: (1) to analyze the differences of carbon released by *R*_*e*_ as well as the dominant environmental variables across the temperate meadow steppe, typical steppe, desert steppe and alpine meadow, respectively; (2) to develop rule-based piecewise regression models to map *R*_*e*_ of diverse grasslands by integrating time-series MODIS products and tower-based observations; and (3) to map the spatial patterns of annual mean *R*_*e*_ for the grassland ecosystems in northern China.

## Methods and materials

### Description of the study area

The study was conducted in the grassland ecosystems of northern China, which is characterized by the arid and semi-arid continental monsoon climate with the highest temperature and rainfall period in summer. Following the east-to-west precipitation gradient, the temperate grasslands in northern China alter longitudinally from meadow steppes in the northeast, through typical steppes in the middle, and to desert steppes in the dry northwest. Alpine meadows are the dominant vegetation type in the Tibetan Plateau zone. These grasslands usually start to grow in early May and wither in late September with the peak biomass in July or August, which provide an important resource for livestock production and global carbon sequestration [5, 33].

**Fig. 1:**
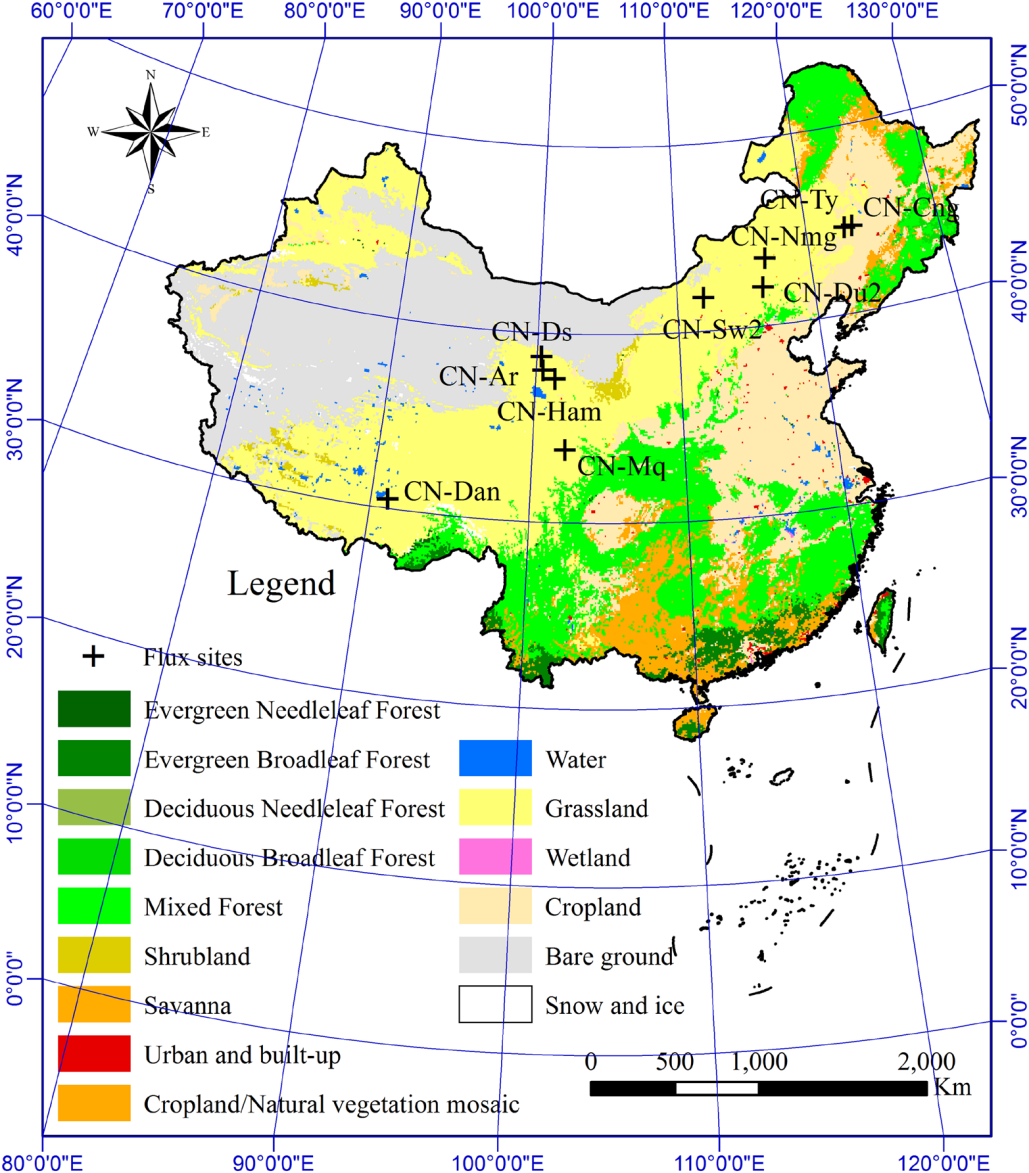
Spatial location and distribution of the grassland flux sites used in the present study. The base map is derived from MODIS land cover product (MCD12Q1 C5, 500 m resolution) based on the International Geosphere Biosphere Programme (IGBP) global vegetation classification scheme in 2010. The detailed descriptions of these flux sites including CN-Cng, CN-Nmg, CN-Du2, CN-Hzz, CN-Sw2, CN-Ham, CN-Ar, CN-Ds, CN-Dan, CN-Ty and CN-Mq can be seen in Table 1

During recent years, a series of EC-based flux towers have been installed by the Coordinated Observation and Synthesis in Arid and Semi-arid China (COSAS), as a part of China Flux Observation and Research Network (ChinaFLUX), which can be used to observe the carbon and water exchanges between the atmosphere and these grasslands in the ecologically fragile areas of northern China (Fig. 1). In total of 11 flux sites are used in the study, which represent the most prevalent types of grassland ecosystems and a wide range of spatial, ecological, and climatic conditions, including two meadow steppe sites, two typical steppe sites, two desert steppe sites and five alpine meadow sites. Detailed descriptions of these flux sites can be found in the associated literature (Table 1).

**Table 1:** Characteristics of the flux sites over grassland ecosystems in northern China

Code	Site name	Location	Temperature (°C)	Precipitation (mm)	Elevation (m)	Vegetation type	Height	Soil type	Years	References
							(m)			
CN-Cng	Changling	44.5934° N	5	400	171	Temperate meadow steppe	0.75	Alkali-saline soil	2007–2010	[56]
		123.5092° E E								
CN-Nmg	Xilinhot^a^	43.5544° N	2	350	1250	Typical steppe	0.4	Chestnut soil	2004	[57]
		116.6714° E								
CN-Du2	Duolun fenced grassland	42.0467° N	1.7	350	1350	Typical steppe	0.45	Chestnut soil	2006–2008	[57]
		116.2836° E								
CN-Hzz	Huazhaizi	38.76519° N	7.5	157	1726	Desert steppe	0.15	Loam	2014–2017	[58]
		100.3186° E								
CN-Sw2	Siziwang	41.7781° N	3.5	283	1438	Desert steppe	0.15	Loam	2011	[59]
		111.8971° E								
CN-Ham	Haibei	37.6127° N	− 1.7	561	3216	Alpine meadow	0.3	Clay loam	2002–2003	[60]
		101.3128° E								
CN-Ar	A’rou	38.0473° N	0.9	403.1	3033	Alpine meadow	0.45	Chestnut soil	2013–2015	[61]
		100.4643°E								
CN-Ds	Dashalong	38.8399° N	− 3.4	319	3739	Alpine meadow	0.15	Chestnut soil	2014–2015	This study
		98.9606° E								
CN-Dan	Dangxiong	30.4978° N	1.3	480	4333	Alpine meadow	0.1	Sandy loam	2004–2005	[62]
		91.0664° E								
CN-Ty	Tongyu degraded grassland^a^	44.5673° N	5.5	345.4	151	Temperate meadow steppe	0.1	Sand soil	2008^b^–2009^b^	[63]
		122.9170° E								
CN-Mq	Maqu	33.8872° N	1.9	593	3443	Alpine meadow	0.25	Silt clay loam	2009^b^	[64]
		102.1407° E								

^a^Represents vegetation type in degraded condition. ^b^Means that only part time of the year’s flux and climate data are measured at this site, generally during the growing season. Among the eleven eddy covariance flux sites, CN-Cng (2010), CN-Ar (2014), CN-Du2 (2008) and CN-Sw2 (2011) are used for validation as a proxy for temperate meadow steppe, Alpine meadow, typical steppe and desert steppe, respectively

### Processing of the EC-based flux data

Both the EC system and the automatic meteorological station were mounted at these grassland sites, which acquired the continuous observations of site-level carbon fluxes (NEE), as well as the relevant climate data, including solar radiation (R_g_), air and soil temperatures (T_a_ and T_s_), relative humidity, soil water content (SWC), precipitation (P) and vapor pressure deficit (VPD). Each EC system was comprised of a three-dimensional sonic anemometer (CSAT3, Campbell Scientific, UT, USA) and a Li-7500 open path CO_2_ and H_2_O gas analyzer (LI-COR Inc., NE, USA). Raw data were continuously recorded at a frequency of 10 Hz on a CR5000 (Campbell Scientific) data logger. The processing procedures including spike detection and despiking, two-dimensional coordinate rotation, time delay removal of H_2_O and CO_2_, virtual temperature correction, density effects (WPL correction) and frequency response corrections were completed using the improved EdiRe software package (developed by the University of Edinburgh) to produce a half-hour flux dataset [39]. However, owing to instrument malfunctions, power failure, and severe weather conditions, approximately 25% of the 1-year observations were lost. Therefore, it was necessary to interpolate these gaps with a standardized gap-filling algorithm. Then, the time-series NEE flux data were partitioned into GPP and *R*_*e*_, separately. In this study, the half-hourly *R*_*e*_ data provided by flux-tower measurements were integrated to the daily time scale, and then averaged over each 8-day period to match the 8-day composite of the MODIS products. The procedures including gap-filling and flux partitioning, were completed using the new R-based package (REddyProc) maintained by the Max Planck Institute for Biogeochemistry [40].

Currently, there are mainly two methods implemented for flux-partitioning: (1) *R*_*e*_ is estimated from the nighttime temperature and extrapolated to daytime [20] and (2) the light–response curve is fit to daytime NEE measurements and *R*_*e*_ is estimated from the intercept of the ordinate [21], which can avoid the use of potentially problematic nighttime data. The latter approach was chosen for flux partitioning because it uses a hyperbolic light-response curve algorithm, modified to account for the temperature dependency of respiration and the VPD limitation of photosynthesis [41]. Including the VPD dependency strongly improved the model’s ability to reproduce the asymmetric diurnal cycle during periods with high VPD, and enhances the reliability of *R*_*e*_ estimates given that the reduction of GPP by VPD may be otherwise incorrectly attributed to higher *R*_*e*_. More details can be seen in the associated references.

### MODIS products and processing

*R*_*e*_ is generally comprised of two sources of respiration: *R*_*a*_ from maintenance respiration and growth respiration, and *R*_*h*_ from rhizomicrobial respiration and microbial decomposition of plant residues and other soil organic matter [42, 31]. Thus, it is strongly affected by plant growth status and climate conditions. This study used the enhanced vegetation index (EVI) and leaf area index (LAI), as well as the land surface water index (LSWI), and mean value of daytime and nighttime temperatures (LST) to represent the vegetation and climate-related variables.

All variables were derived from the time-series MODIS data, which can avoid the complications and difficulties associated with merging disparate data sources. The 8-day land surface reflectance (MOD09A1, V6, with resolution of 500 m), LAI product (MOD15A2, V6, with resolution of 500 m) and LST data (MOD11A2, V6, with resolution of 1 km) were downloaded from the NASA’s Earth Observing System Data and Information System (https://search.earthdata.nasa.gov). We only used the data described as good quality in the quality layer. These remote sensing-based products were re-sampled to a spatial resolution of 1 km, and the data where corresponded to the geographical location of each flux site were extracted for model development. In addition, the vegetation type map at 1 km resolution in 2010 was obtained from Nanjing Institute of Geography and Limnology, Chinese Academy of Science. The product specifically classified grasslands in China into temperate meadow steppe, typical steppe, desert steppe and alpine meadow, which can meet the needs to estimate *R*_*e*_ across different grassland types in this study. Then, all these data were used to develop models and map the spatio-temporal patterns of *R*_*e*_ in grassland ecosystems in northern China.

### Statistical analyses

To reveal the dominant environmental factors controlling the variability in *R*_*e*_ on an 8-day time scale over the whole year and different phenological periods (growing season *vs* dormant season) at these four different grassland ecosystems, the Pearson correlation coefficient (*r*) was calculated to examine the relationships between site-level *R*_*e*_ observations and these vegetation and climate-related variables. As these grasslands usually start to grow in early May and wither in late September, the growing period and dormant period were defined from WOY 16 to 34 and the rest of 1 year (WOY 1 to 15, and WOY 35 to 46), respectively. Then, we aimed to develop a rule-based piecewise regression model to capture the seasonal variations in *R*_*e*_ of temperate meadow steppe, typical steppe, desert steppe and alpine meadow in northern China. For each grassland type, the training set and test set of these flux data were instructed in Table 1. The study defined the model developed directly from the entire year as model 1, and the model from the growth period and dormant season as model 2. The model’s accuracy was evaluated using two widely-used indicators: *R*^*2*^ and the root-mean-square error (RMSE). The best model generally had the highest *R*^*2*^ and lowest RMSE values. Finally, the optimal models were used to map the spatial patterns of annual mean *R*_*e*_ for the grasslands in northern China. All statistical analyses were performed using SPSS 19.0 (IBM, Chicago, IL, USA). In addition, the MODIS Reprojection Tool (MRT) and the Interactive Data Language (IDL) in ENVI 5.3 were used to process over 2000 scenes of MODIS data and large-area estimation.

## Results

### Differences of annual mean R_e_ across grasslands

**Fig. 2:**
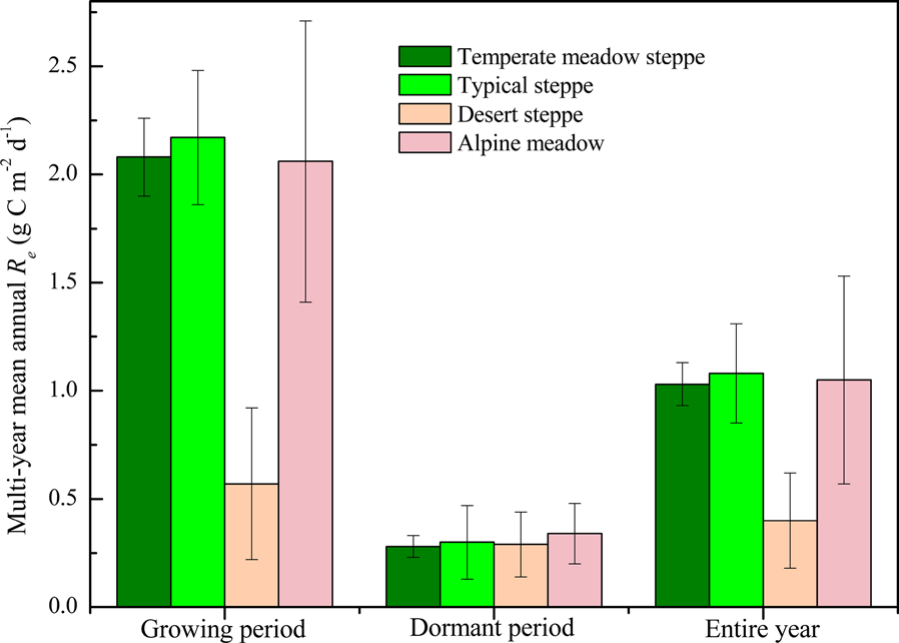
Comparisons of multi-year mean *R*_*e*_ during the growing period, dormant period and the entire year across grasslands in northern China. Error bars represent one standard error

Multi-year mean *R*_*e*_ of the temperate meadow steppe, typical steppe, desert steppe and alpine meadow ecosystems in northern China were exhibited in Fig. 2 with apparent differences in magnitude. It revealed that C emissions by *R*_*e*_ were mainly concentrated in the growing season, which was even about twice and seven times of *R*_*e*_ during the dormant period for desert steppe and the other grasslands, respectively. The low *R*_*e*_ of desert steppe throughout the year can be ascribed to relatively sparse vegetation coverage. This study also found that during non-growing season, only small differences in *R*_*e*_ existed among these grassland types. Generally, typical steppe exhibited the strongest *R*_*e*_ of 1.08 ± 0.23 g C m^−2^ d^−1^, followed by temperate meadow steppe (1.03 ± 0.10 g C m^−2^ d^−1^) and alpine meadow (1.05 ± 0.48 g C m^−2^ d^−1^), and undoubtedly desert steppe had the weakest *R*_*e*_. These analyses again emphasized the importance to quantify the patterns of grassland *R*_*e*_ separately for large-area estimation.

### Seasonal variations in R_e_ and environmental controls

**Fig. 3:**
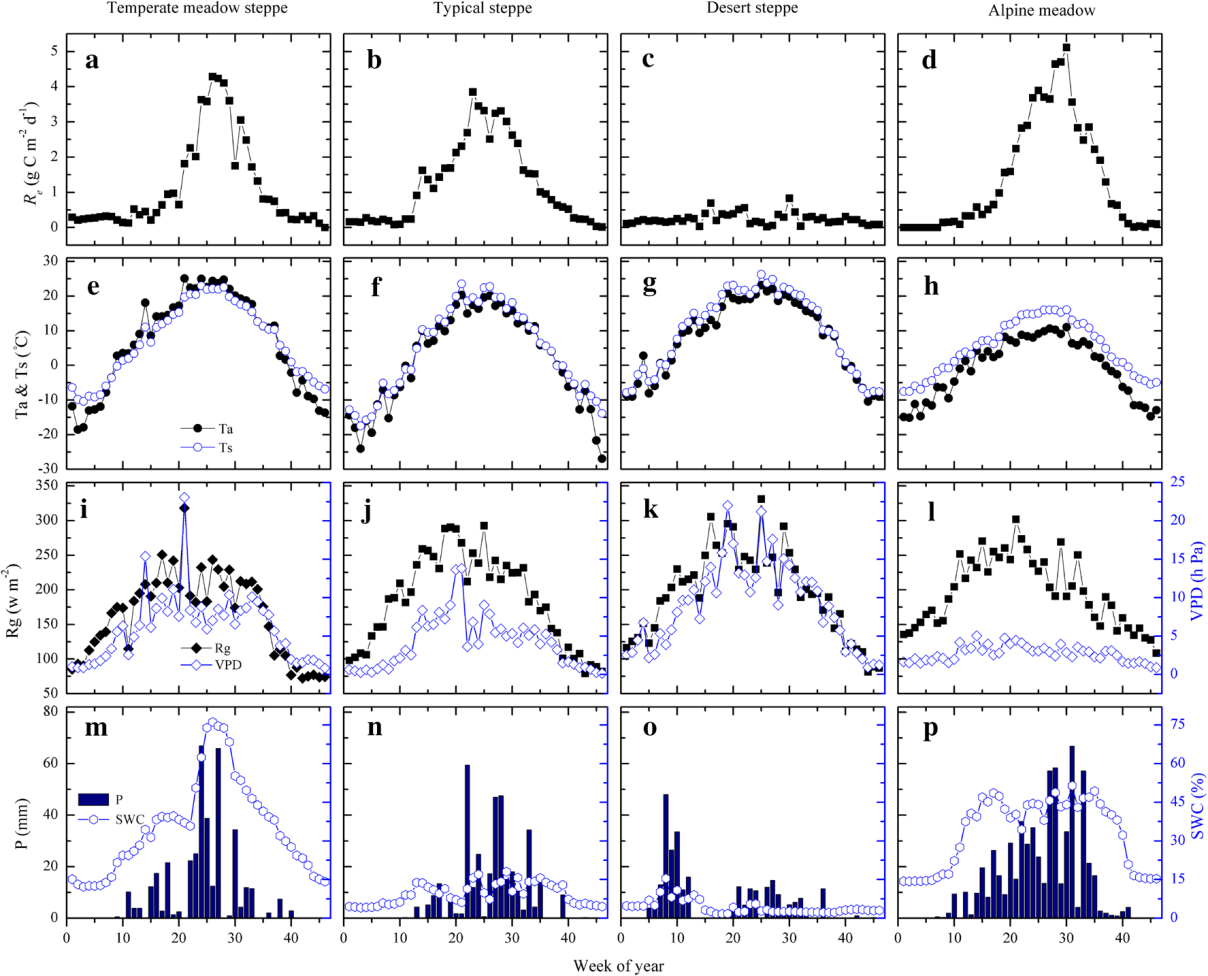
Seasonal trends of **a**–**d** ecosystem respiration (*R*_*e*_), **e**–**h** air temperature (T_a_) and soil temperature (T_s_), **i**–**l** solar radiation (R_g_) and vapor pressure deficit (VPD), **m**–**p** precipitation (P) and soil water content (SWC) in an 8-day interval at the representative grassland flux sites for selected years including CN-Cng 2008 (Temperate meadow steppe), CN-Nmg 2004 (Typical steppe), CN-Hzz 2014 (Desert steppe) and CN-Ham 2003 (Alpine meadow)

**Fig. 4:**
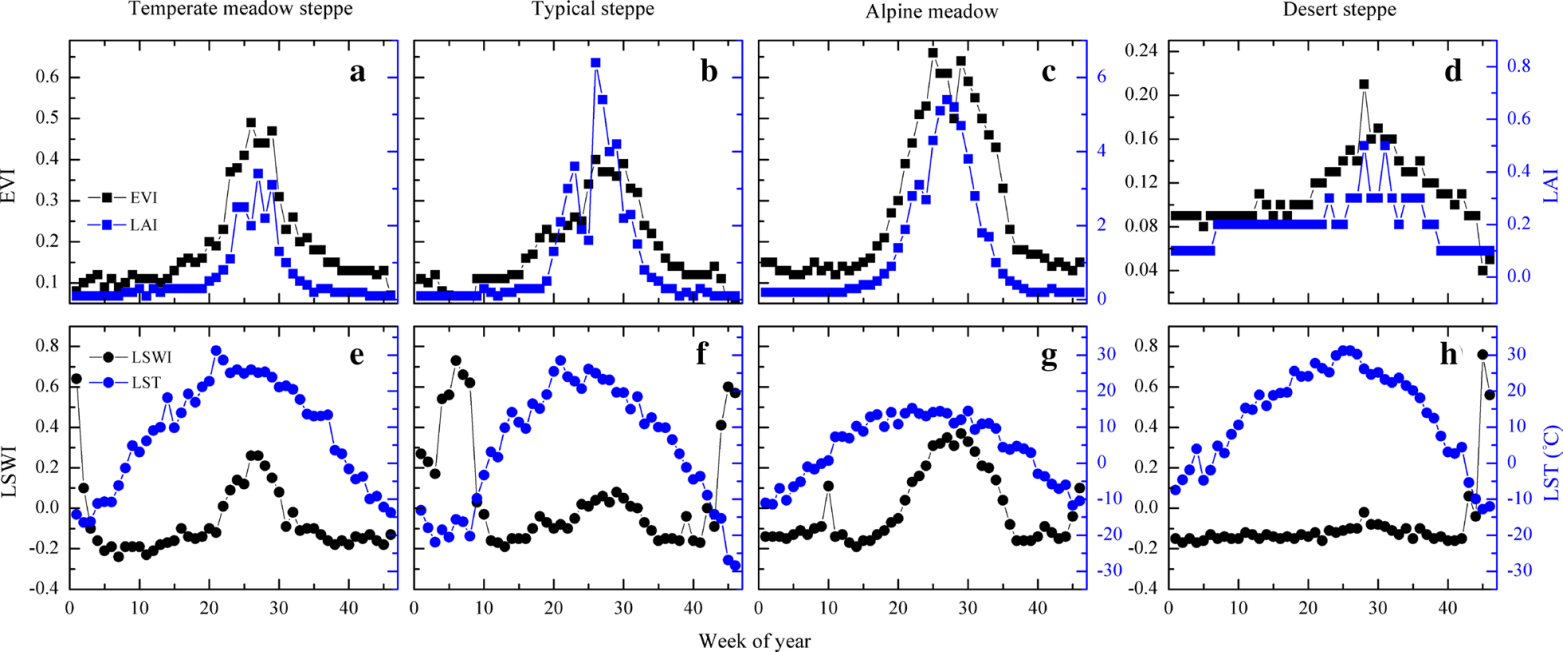
The seasonal dynamics of 8-day EVI, LAI, LSWI and LST at the representative grassland flux sites for selected years including CN-Cng 2008 (Temperate meadow steppe), CN-Nmg 2004 (Typical steppe), CN-Hzz 2014 (Desert steppe) and CN-Ham 2003 (Alpine meadow)

Seasonal dynamics in *R*_*e*_ as well as the vegetation and climate-related variables across the four grassland ecosystems were illustrated in Figs. 3 and 4. It implied that except the desert steppe site (CN-Hzz), *R*_*e*_ of the other three grassland types exhibited an apparent single-peak pattern. As the temperature rose in spring, *R*_*e*_ gradually increased with plant growth, and reached the peak in July or August. However, the peak periods came earlier in typical steppe (CN-Nmg) at about week of year–WOY 23, followed by temperate meadow steppe (CN-Cng, WOY 25) and alpine meadow (CN-Ham, WOY 30). Natural rainfall generally occurred in summer with large fluctuations around the year. Contrastingly, relatively smooth SWC can reflect the true water availability. As a proxy of vegetation response to environmental variables, EVI and LAI exhibited consistent trends as *R*_*e*_. Several periods of the remote-derived LSWI in winter were quite large due to the snow cover on and under the grasslands.

Table 2 revealed that almost all variables were strongly correlated to *R*_*e*_ without considering the phenology information. However, during the dominant growth period for respiration in temperate meadow steppe, typical steppe and alpine meadow, only temperature (T_a_ and T_s_) and SWC strongly and positively affected the variability in *R*_*e*_. In the desert steppe, *R*_*e*_ was found to be exerted a strong effect by SWC across growing and dormant seasons, which highlighted the water condition rather than temperature as the most important controlling factor in the extremely dry ecosystems. All the remotely-sensed vegetation indexes and climate-related LST and LSWI exhibited strong correlations, implying great potential to quantify the variability in *R*_*e*_ using RS technique.

**Table 2:** Pearson correlation analysis between 8-day *R*_*e*_ and the controlling environmental factors across the four grassland ecosystem types

Grassland type	Period	R_g_ (W/m^2^)	T_a_ (°C)	T_s_ (°C)	VPD (h Pa)	P (mm)	SWC (%)	EVI	LAI	LSWI	LST (°C)
Temperate meadow steppe	Growing	− 0.115	*0.538*^*a*^	*0.613*^*a*^	− 0.287^b^	0.252^b^	*0.351*^*a*^	*0.745*^*a*^	*0.692*^*a*^	*0.704*^*a*^	*0.371*^*a*^
	Dormant	*0.411*^*a*^	*0.707*^*a*^	*0.714*^*a*^	*0.685*^*a*^	0.231^b^	*0.625*^*a*^	*0.378*^*a*^	*0.635*^*a*^	− 0.269^b^	0.689^a^
	Entire year	*0.570*^*a*^	*0.749*^*a*^	*0.786*^*a*^	*0.520*^*a*^	*0.459*^*a*^	*0.669*^*a*^	*0.869*^*a*^	*0.851*^*a*^	0.093	0.721^a^
Typical steppe	Growing	0.073	*0.760*^*a*^	*0.707*^*a*^	− 0.149	*0.568*^*a*^	*0.495*^*a*^	*0.721*^*a*^	*0.644*^*a*^	*0.748*^*a*^	*0.669*^*a*^
	Dormant	*0.506*^*a*^	*0.554*^*a*^	*0.553*^*a*^	*0.579*^*a*^	0.269^b^	*0.571*^*a*^	*0.377*^*a*^	*0.451*^*a*^	− 0.235	*0.532*^*a*^
	Entire year	*0.644*^*a*^	*0.776*^*a*^	*0.803*^*a*^	*0.581*^*a*^	*0.700*^*a*^	*0.644*^*a*^	*0.861*^*a*^	*0.794*^*a*^	− 0.011	*0.759*^*a*^
Desert steppe	Growing	0.138	0.171	0.261	0.193	0.197	*0.538*^*a*^	*0.499*^*a*^	*0.621*^*a*^	0.338^b^	0.110
	Dormant	0.086	0.175	0.225	0.056	− 0.119	*0.611*^*a*^	0.296^b^	0.052	− 0.262	0.175
	Entire year	*0.370*^*a*^	*0.408*^*a*^	*0.449*^*a*^	*0.421*^*a*^	0.101	*0.475*^*a*^	*0.542*^*a*^	*0.653*^*a*^	− 0.198	*0.392*^*a*^
Alpine meadow	Growing	− 0.224^b^	*0.535*^*a*^	*0.531*^*a*^	0.039	0.142	*0.397*^*a*^	*0.764*^*a*^	*0.382*^*a*^	*0.739*^*a*^	0.281^b^
	Dormant	0.275^b^	*0.652*^*a*^	*0.628*^*a*^	*0.379*^*a*^	*0.445*^*a*^	*0.630*^*a*^	*0.577*^*a*^	*0.566*^*a*^	− 0.206^b^	*0.561*^*a*^
	Entire year	0.265^b^	*0.629*^*a*^	*0.647*^*a*^	*0.325*^*a*^	*0.484*^*a*^	*0.565*^*a*^	*0.837*^*a*^	*0.548*^*a*^	*0.326*^*a*^	*0.531*^*a*^

^a^ and ^b^ mean that correlations are significant at the 0.01 level and 0.05 level, respectively. R_g_, T_a_, T_s_, VPD, P and SWC are observed at the flux tower sites listed in Table 1. Time-series EVI, LAI, LSWI and LST data are derived from the corresponding MODIS products. All these data are at an 8-day interval

### Model development for quantitative remote sensing

**Fig. 5:**
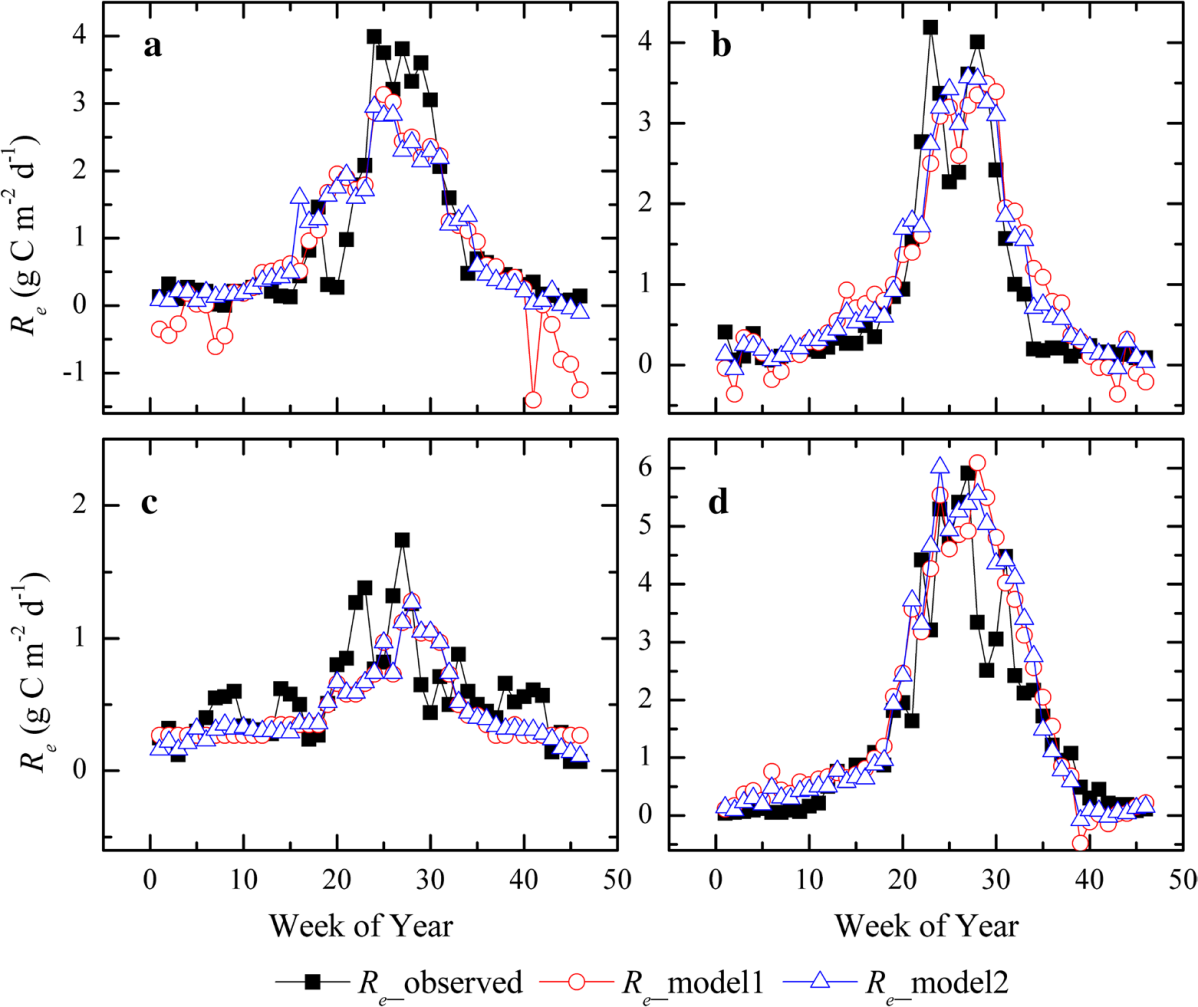
Performance of the remotely-derived *R*_*e*_ (*R*_*e*__model1 and *R*_*e*__model2) in capturing seasonal variations of tower-based *R*_*e*_ (*R*_*e*__observed) at the temperate meadow steppe (**a**), typical steppe (**b**), desert steppe (**c**) and alpine meadow (**d**) sites, respectively

By integrating site-level *R*_*e*_ observations and time-series MODIS products, the study developed the optimal models for capturing the variability in *R*_*e*_ of different grasslands using the stepwise multiple regression method (Table 3). Mainly two models were proposed with good performances. Generally, the model 2 established from the growth period and dormant season separately had relatively higher accuracy in estimating *R*_*e*_ than that directly from the whole year (model 1). Besides the desert steppe, *R*^*2*^ and RMSE of the other grasslands varied from 0.77 to 0.88 and from 0.41 to 0.67 g C m^−2^ d^−1^, respectively. The model with only LAI as an explanatory variable also got good estimates for the desert steppe. Figure 5 further certified that the model promised well to estimate 8-day *R*_*e*_ and captured the broad trend of seasonal patterns, especially for the *R*_*e*__model 2, whereas the *R*_*e*__model 1 caused individual abnormal *R*_*e*_ estimates in wintertime. In spite of good accuracy, the RS-based models remained lacking of skills to capture abrupt changes in *R*_*e*_ during the summertime growing season.

**Table 3:** Regression analysis of 8-day *R*_*e*_ and the associated MODIS-derived products across the four grassland ecosystems using the stepwise multiple regression method

Grassland type	Period	Regression models					Training set		Test set	
		Constant	EVI	LAI	LSWI	LST	*R*^*2*^	RMSE	*R*^*2*^	RMSE
Temperate meadow steppe	Growing	1.088	4.078	–	3.743	–	0.57	0.72	0.77	0.45
	Dormant	0.064	2.362	–	0.268	0.014	0.61	0.13		
	Entire year	− 0.619	8.487	–	–	0.018	0.80	0.51	0.71	0.63
Typical steppe	Growing	0.561	–	–	6.058	0.096	0.72	0.60	0.88	0.41
	Dormant	0.044	3.463	–	0.659	0.021	0.41	0.26		
	Entire year	− 0.083	5.633	–	1.419	0.040	0.84	0.49	0.81	0.50
Desert steppe	Growing	0.213	–	0.758	–	–	0.39	0.32	0.50	0.21
	Dormant	0.122	2.035	–	–	− 0.003	0.11	0.17		
	Entire year	0.190	–	0.775	–	–	0.43	0.25	0.45	0.27
Alpine meadow	Growing	− 0.321	7.198	− 0.208	2.854	–	0.73	0.98	0.85	0.67
	Dormant	− 0.264	3.826	0.897	0.804	0.019	0.65	0.20		
	Entire year	− 0.520	6.947	–	1.318	0.020	0.81	0.67	0.83	0.69

Time-series EVI, LAI, LSWI and LST data are derived from the corresponding MODIS products. All data are at an 8-day interval. The training set and test set of these flux sites are instructed in Table 1. When using the stepwise multiple regression method to develop models, only the best is exhibited here. The model’s performance was evaluated using the site-level flux measurement

**Fig. 6:**
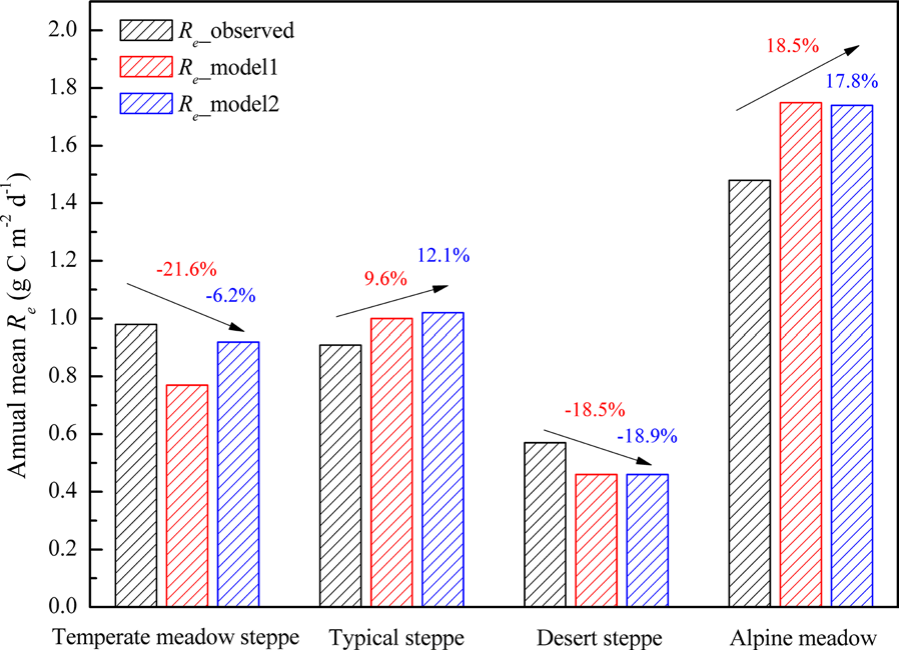
Comparisons of annual mean *R*_*e*_ observations and the remotely-derived products across the temperate meadow steppe, typical steppe, desert steppe and alpine meadow sites

Meanwhile, this study evaluated the model’s performance on annual mean *R*_*e*_ across these four grasslands. Figure 6 revealed that the remotely-derived products slightly underestimated *R*_*e*_ at the temperate meadow steppe and desert steppe sites, but overestimated *R*_*e*_ at the typical steppe and alpine meadow sites. The *R*_*e*__model1 had superior *R*_*e*_ estimates with mean bias of 9.6% at the typical steppe site, while *R*_*e*__model2 only had an underestimation of 6.2% at the temperate meadow steppe site. Both models had similar accuracy at the desert steppe and alpine meadow sites.

### Spatial patterns of satellite-derived R_e_

**Fig. 7:**
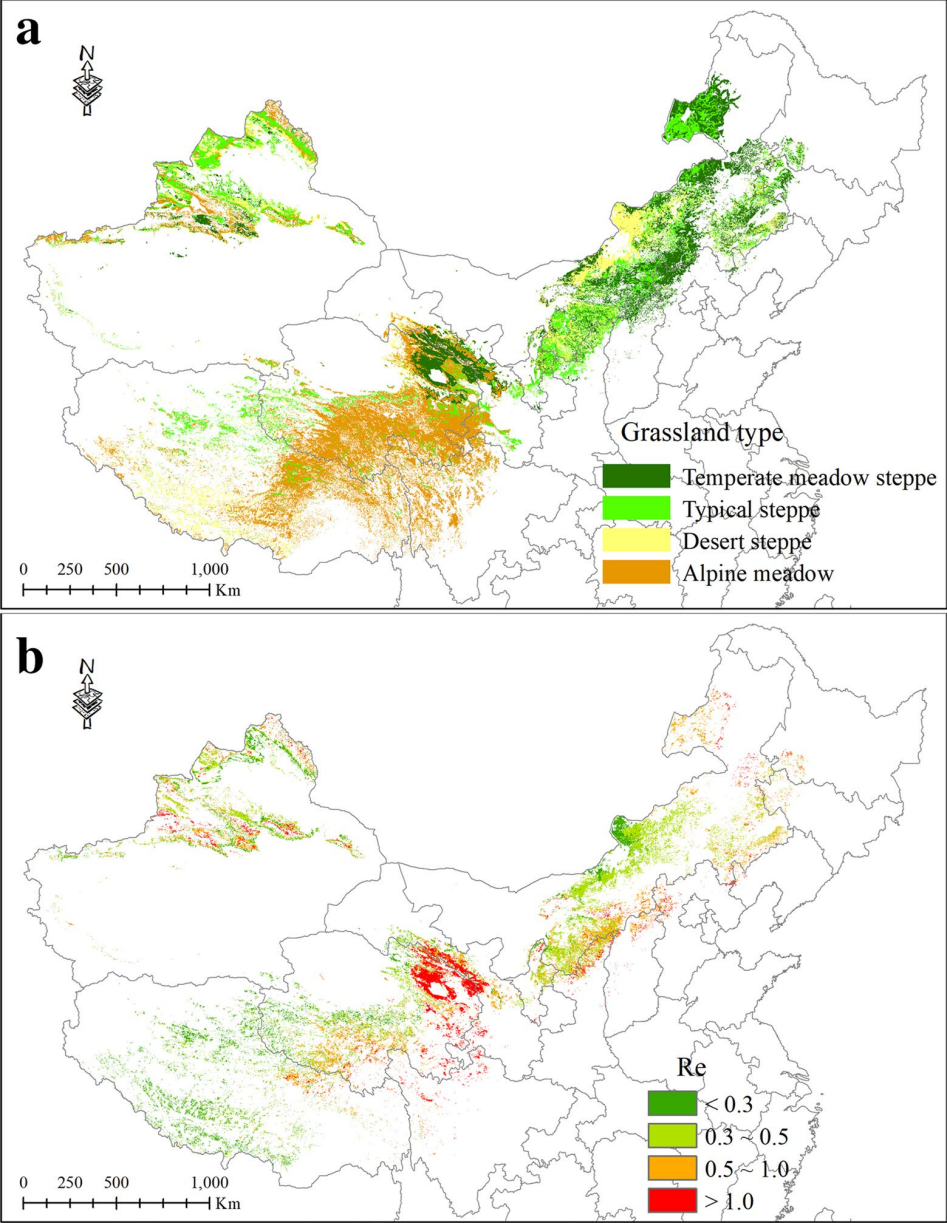
Spatial patterns of grassland types (**a**) and annual mean *R*_*e*_ (**b**) over the northern China’s grasslands in 2010. The unit of *R*_*e*_ is g C m^−2^ d^−1^

The study mapped the spatial distribution of annual mean *R*_*e*_ for grasslands in northern China using the proposed regression models (Fig. 7), which were extrapolated from the 8-day *R*_*e*_ estimates throughout the year of 2010. Generally, our results showed a declining trend from the southeast to the northwest direction. The highest *R*_*e*_ usually occurred in the temperate meadow steppe under good hydrothermal conditions. However, the desert steppe and the Tibetan alpine meadow emitted relatively fewer CO_2_ through respiration. The total regional *R*_*e*_ estimate in the northern China’s grasslands during 2010 was about 100.66 Tg C, which was approximately 1/3 of the carbon uptake through plant photosynthesis with 332.08 Tg C from the MODIS GPP product. Specially, the ratio of *R*_*e*_ to GPP exhibited the highest value in the desert steppe (88.6%), followed by the temperate meadow steppe (36.9%) and typical steppe (21.5%), with the lowest ratio in the alpine meadow of 18.0%.

## Discussion

Along the east–west precipitation gradient in northern China, a wide variety of grasslands constitute the dominant landscape in servicing the ecological environment and socio-economics of the region and in supporting diverse species of plants and animals [32]. The meadow steppes usually occur in the most moist and fertile sites among the four grassland ecosystem types, typically in areas with annual precipitation of about 450 mm. The typical steppes are developed under a semi-arid climate in the temperate zone with annual precipitation of around 350 mm and the plant species are characteristically drought-tolerant. The desert steppes are the most arid grassland type, occurring in areas with annual precipitation between 150 and 250 mm under the influence of continental climate. Alpine steppes are found between 2300 and 5300 m a.s.l. in southwestern China. The common construction plant species are cold- and drought-tolerant grasses and small shrubs. Arid and semiarid ecosystems are often overlooked in this regard because of their spatially sparse vegetation and fragile environmental conditions [43, 44]. In fact, the grassland ecosystems are not only economically important, but recent work has shown water-limited systems to drive the interannual variability in the global C cycle significantly [45, 46]. Therefore, a deep understanding of the C sequestration potential as well as environmental controls over the northern China’s grasslands is vital for reducing the uncertainty in future climate projections given the large area of grasslands.

Accurate quantification of the main C fluxes across terrestrial ecosystems is crucial to our understanding of global carbon balance [23, 47]. Till now, there are fewer successful models of *R*_*e*_ compared to GPP particularly using the RS data directly [29, 48]. Reichstein et al. [49] found that soil water and temperature were good predictors for *R*_*s*_, and adding LAI as a proxy for productivity further improved the accuracy. This study was based on closed-chamber data from forest and shrubland sites across Europe and North America, but it was suggested that the variables could easily be acquired from RS data. Anderson et al. [50] used a model which calculated soil moisture from microwave sensing, soil temperature from thermal imaging, and LAI, showed good agreement with tower flux data over pasture land in Oklahoma. However, model development over such a small area is unlikely to generate a model which is applicable to other ecosystems or climates, and more validation work is also needed. In spite of this, subsequent studies have suggested that the strong relationships between *R*_*s*_ and *R*_*e*_ and GPP [26], LST [17, 25, 48] from the time-series RS data.

Changes in temperature and water availability, and thus climate change, are likely to be significant drivers of the future C balance of land systems and their feedbacks to climate change. Grasslands in the arid and semi-arid regions are ecologically fragile and sensitive to climate change and human disturbances, especially to the changes in precipitation [51]. The precipitation in arid and semi-arid regions is highly variable both temporally and spatially. Fluctuations in carbon budget have been found to be closely associated with interannual and intra-annual variations in precipitation in arid and semi-arid ecosystems, and persistent drought has caused a general decrease in vegetation productivity in the grassland systems of northern China [5]. These work also demonstrated that water condition is an important environmental indicator for the estimates of *R*_*e*_ in the grassland ecosystems. Soil moisture affects *R*_*e*_ processes in various ways, including the growth and development of both aboveground vegetation and roots, growth and activity of microbial populations, in addition to gas transport throughout soils [52, 53]. Green et al. [2] also emphasize that the capacity of continents to act as a future carbon sink critically depends on the response of C fluxes to soil moisture. The study used the LSWI derived from the shortwave infrared (SWIR) and the near infrared (NIR) bands of MODIS data to represent land surface water condition, and got reasonably good performance. Actually, LSWI is different from soil moisture (SWC) because most information of LSWI reflects the total amount of liquid water in vegetation [54]. An interesting avenue of future work would be to consider employing the soil moisture data from the Advanced Microwave Scanning Radiometer-Earth Observing System (AMSE-E) [55]. The problem with many of these studies, however, is that they are too narrow for comparison. They consider one particular site in one particular ecosystem, or attempt to create a global model often focus on a narrow range of only four or five ecosystem types [17, 29]. Grasslands and their huge variety of types are almost never included as a separate category in remote sensing models of carbon flux, and as such are certain to be over or under-estimated. Ge et al. [30] proposed similar models to evaluate grassland *R*_*e*_ at a regional scale by integrating flux measurements and the corresponding MODIS products. But this study simplified the grasslands in northern China by alpine grasslands and temperate grasslands. In fact, large differences existed among the temperate meadow steppe, typical steppe and desert steppe when exploring the dominant environmental controls and developing associated models. Meanwhile, the variability in *R*_*e*_ rely on grassland types and local environmental conditions. Therefore, our research on remotely monitoring *R*_*e*_ from various grasslands along a large-scale east–west transect across northern China can provide more information than previous studies.

Our results suggested that simple models relying entirely on spatial data have the potential to estimate *R*_*e*_ over the diverse grassland ecosystems. This result provides a framework for the development of *R*_*e*_ models aimed to obtain spatial pattern in *R*_*e*_. Meanwhile, the models incorporating the phenology information generally provided better estimations. In this study, we simply separated the growing season and dormant season as these grasslands usually start to grow in early May and wither in late September. To acquire more specific phenological information of each pixel, the global land surface phenology metrics at yearly intervals (MCD12Q2) can be used for auxiliary analysis. But the models would be complicated for large-scale extrapolation owing to strong spatial heterogeneity. In addition, there is a need for more long-term studies in order to monitor the interannual variations of *R*_*e*_ as well as the underlying mechanisms. The study calculated the spatial distribution of grassland *R*_*e*_ in 2010 because we only have a period of classification data with detailed grassland types. The total of carbon emissions of *R*_*e*_ across the northern China’s grasslands was accumulated to 100.66 Tg C during 2010, which was approximately 1/3 of the regional carbon uptake through plant photosynthesis with 332.08 Tg C from the MODIS GPP product. Particularly, the carbon use efficiency was the highest in the alpine meadow, followed by the typical steppe, meadow steppe and desert steppe. However, this approach generally need large amounts of *R*_*e*_ data to constrain parameters. The regression coefficients obtained at this study would not work well for other grassland sites with different climate, soil, and vegetation. Although we tried to establish the robust models for the temperate meadow steppe, typical steppe, desert steppe and alpine meadow using 11 flux sites, the representativeness of these limited sites presumably affected the general applicability of our predictive model. Thus, the current Coordinated Observation and Synthesis in Arid and Semi-arid China should be augmented by building more sites across a full range of grassland types.

Conclusion

On the basis of 24 EC-based years of flux measurements over 11 grasslands sites under a wide range of geographic, weather and ecological conditions, the study was an attempt to upscale site-level *R*_*e*_ data to the northern China’s grasslands including temperate meadow steppe, meadow steppe, desert steppe and alpine meadow, using the satellite-based RS data. The results demonstrated that the rule-based piecewise regression models can successfully estimate the seasonal variations in *R*_*e*_ and provide a new framework to map the regional patterns of large-scale *R*_*e*_. The reduction of uncertainties in *R*_*e*_ is crucial for projecting climate change impacts on terrestrial carbon cycling and future atmospheric CO_2_ concentrations. This work also offers an opportunity to further understand the environmental drivers controlling the variability in *R*_*e*_. Specially, not only temperate but also soil water content, had a strong correlation with grassland *R*_*e*_, which should not be neglected when developing the RS models for arid and semiarid ecosystems. Meanwhile, the models incorporating the phenology information generally performed better. The spatial patterns of *R*_*e*_ across the northern China’s grasslands exhibited a distinctly declining trend from the southeast to the northwest, with a regional estimate of approximately 100.66 Tg C during 2010. With more periods of grassland classification information, future studies can even evaluate long-term land-use change and its impact on the large-scale *R*_*e*_ of grasslands.

## Data Availability

The data used in this article are available upon request.

## Abbreviations

*R*_*e*_: Ecosystem respiration
GPP: Gross primary production
MODIS: Moderate-resolution Imaging Spectroradiometer
*R*_*a*_: Autotrophic respiration
*R*_*h*_: Heterotrophic respiration
EC: Eddy covariance
RS: remote sensing
*R*_*s*_: Soil respiration
LST: Land surface temperature
NDVI: Normalized difference vegetation index
EVI: Enhanced vegetation index
LAI: Leaf area index
*R*^*2*^: Coefficient of determination
RMSE: Root-mean-square error
*r*: Correlation coefficient
ChinaFLUX: China Flux Observation and Research Network
NEE: Net ecosystem carbon flux
R_g_: Solar radiation
T_a_: Air temperature
T_s_: Soil temperature
SWC: Soil water content
P: Precipitation
VPD: Vapor pressure deficit
LSWI: Land surface water index
